# Supplementary material for: Relationship between Fundamental Movement Skills and Physical Activity in Preschool-aged Children: A Systematic Review
Source: Int J Environ Res Public Health. 2020 May 19;17(10):3566. doi: 10.3390/ijerph17103566 (PMC7277928; doi:10.3390/ijerph17103566)
Supplement: Supplementary file 1 [file ijerph-17-03566-s001.pdf]

Table S1. Search strategies used for each database

**Database**: Web of Science **Number of results**: 357<br>TS=(preschool* OR kindergarten* OR "early child*" OR "young child*") AND TS=("motor skill*" OR "movement skill*" OR "motor ability*" OR "movement ability*" OR "motor competence" OR "movement competence" OR "motor performance" OR "movement performance" OR "motor proficiency" OR "movement proficiency" OR locomotor OR "object control" OR manipulat* OR stability*) AND TS=("physical activit*") AND TS=(associate* OR relat* OR predict* OR indict* corelat* OR effect*)<br>**TS**=topic **Language**: English **Document types**: Article
**Database**: PubMed **Number of results**: 201<br>All Field: (preschool* OR kindergarten* OR "early child*" OR "young child*") AND ("motor skill*" OR "movement skill*" OR "motor ability*" OR "movement ability*" OR "motor competence" OR "movement competence" OR "motor performance" OR "movement performance" OR "motor proficiency" OR "movement proficiency" OR locomotor OR "object control" OR manipulat* OR stability*) AND ("physical activit*") AND (associate* OR relat* OR predict* OR indict* corelat* OR effect*)<br>**Language**: English **Article types**: article
**Database**: ScienceDirect **Number of results**: 639<br>**Find articles with these terms**: (preschool* OR kindergarten* OR "early child*" OR "young child*") AND ("motor skill*" OR "movement skill*" OR "motor ability*" OR "movement ability*" OR "motor competence" OR "movement competence" OR "motor performance" OR "movement performance" OR "motor proficiency" OR "movement proficiency" OR locomotor OR "object control" OR manipulat* OR stability*) AND ("physical activity") AND (associate* OR relat* OR predict* OR indict* corelat* OR effect*)<br>**Article type**: article
**Database**: EBSCO (SPORTDiscus, ERIC, Academic Search Premier) **Number of results**: 82<br>AB=(preschool* OR kindergarten* OR "early child*" OR "young child*") AND SU=("motor skill*" OR "movement skill*" OR "motor ability*" OR "movement ability*" OR "motor competence" OR "movement competence" OR "motor performance" OR "movement performance" OR "motor proficiency" OR "movement proficiency" OR locomotor OR "object control" OR manipulat* OR stability*) AND SU=("physical activit*") AND AB=(associate* OR relat* OR predict* OR indict* corelat* OR effect*)<br>AB=abstract SU=subject **Language**: English **Document types**: Article
**Database:** Cochrane Library **Number of results:** 94<br>Title Abstract Keywords=(preschool* OR kindergarten* OR "early child*" OR "young child*") AND Title Abstract Keywords =("motor skill*" OR "movement skill*" OR "motor ability*" OR "movement ability*" OR "motor competence" OR "movement competence" OR "motor performance" OR "movement performance" OR "motor proficiency" OR "movement proficiency" OR locomotor OR "object control" OR manipulat* OR stability*) AND Title Abstract Keywords =("physical activity*") AND Title Abstract Keywords=(associate* OR relat* OR predict* OR indict* corelat* OR effect*)<br>**Language: English** **Document types**: trial
